# Heat Stress Induces Partial Resistance to Tomato Bushy Stunt Virus in *Nicotiana benthamiana* Via Combined Stress Pathways

**DOI:** 10.3390/v17091250

**Published:** 2025-09-16

**Authors:** Nurgul Iksat, Almas Madirov, Dana Artykbayeva, Oleksiy Shevchenko, Kuralay Zhanassova, Zhaksat Baikarayev, Zhaksylyk Masalimov

**Affiliations:** 1 Rustem Omarov Plant Biotechnology Laboratory, Department of Biotechnology and Microbiology, L.N. Gumilyov Eurasian National University, Astana 010008, Kazakhstan; 2 Department of Virology, ESC “Institute of Biology and Medicine”, Taras Shevchenko National University of Kyiv, 01601 Kyiv, Ukraine

**Keywords:** plant virus, combined stress, ROS, heat stress, TBSV

## Abstract

Global climate change is the impact of combined abiotic and biotic stresses negatively affecting plant health and productivity. This study investigated the molecular and cellular responses of *Nicotiana benthamiana* L. plants to wild-type tomato bushy stunt virus (wtTBSV) infection under conditions of pre-existing heat stress. The experiments were conducted under controlled temperature regimes of 30 °C and 37 °C in combination with virus challenge. Morphological and biochemical analyses in plants under the influence of combined stress showed the alleviation of disease symptoms, reduction in virus content and reduced expression levels of viral proteins P19 and P33. Under conditions of combined stress, accumulation of hydrogen peroxide and malondialdehyde, as well as activation of the antioxidant enzyme catalase, especially in root tissues, were observed. Notably, at 37 °C, virus infection was suppressed despite high levels of oxidative stress, whereas at 30 °C, a marked decrease in the expression of host factors was observed. The results indicate that thermal stress modulates virus–host interactions and activates defense mechanisms, including antioxidant and RNA interference pathways. Therefore, temperature adaptation can be considered as a promising strategy for enhancing plant resistance to viral pathogens under climate changes.

## 1. Introduction

Modern agriculture operates under the constant interplay of multiple biotic and abiotic stress factors, a situation increasingly aggravated by the ongoing shifts associated with global climate change. Increasingly higher mean annual temperatures, growing frequency of extreme weather events, and alterations in seasonal cycles profoundly affect plant growth, development, and immune competence. These environmental pressures not only alter the balance between host and pathogen but also create favorable conditions for changes in the occurrence and severity of phytopathological processes, with viral diseases standing out as a particularly serious threat to global food security [1,2].

The influence of temperature on the course of viral infections in plants is complex and often bidirectional. In certain cases, elevated temperatures favor virus replication and suppress host’s immune responses; in others, they may trigger defense pathways such as RNA interference (RNAi) [3,4]. While the impact of individual abiotic stressors, such as drought, salinity, or sudden temperature shifts, has been examined in detail, plant responses to the action of combined stresses remain far less understood, despite being far more typical for the real-world agricultural conditions. Such combined stresses often induce intricate cross-regulatory signaling networks that cannot be easily inferred from single-stress studies [5,6]. Among plant pathogens shown to be responsive to temperature variations, viruses of the *Tombusviridae* family, particularly *Tombusvirus lycopersici*, require special attention. The family *Tombusviridae* consists of 16 genera, which are divided into three subfamilies (*Procedovirinae*, *Regressovirinae*, and *Calvusvirinae*) and have an icosahedral viral capsid, except for Dianthovirus [7]. TBSV has a wide host range and causes diverse plant symptoms ranging from asymptomatic infection to severe leaf mosaic, deformation and even necrosis [8,9,10]. Its ~4.8 kb positive-sense RNA genome encodes five major proteins: replication proteins (P33 and P92), coat protein (CP41), movement protein (P22), and RNAi suppressor (P19) [11,12,13,14]. P33 orchestrates the assembly of replication complexes, CP41 not only serves as a structural component but also facilitates systemic transport of the viral ribonucleoprotein complex (RNPc) by interacting with the cytoskeleton and cellular membranes, while P19 neutralizes the plant’s RNAi defense by binding virus-derived small interfering RNAs (vsiRNAs) and blocking their incorporation into the RISC complex [15].

Like many other plant viruses, TBSV recruits host cellular proteins to optimize its replication. Heat shock proteins HSP70 and HSP90 are among the most important of these proviral factors. HSP70 mediates the recruitment of viral and host proteins to replication sites, while HSP90 stabilizes the viral RNA-dependent RNA polymerase P92 and promotes the formation of functional replication complexes [16]. The fact that both HSP70 and HSP90 are themselves induced by elevated temperatures [17] adds an additional layer of complexity, as the same thermal conditions can either accelerate or hinder viral infection depending on the interplay between proviral and antiviral processes [18,19].

A central feature of the plant response to both biotic and abiotic stress is the activation of reactive oxygen species (ROS) signaling networks [20]. The oxidative burst, characterized by a rapid and transient increase in the levels of hydrogen peroxide (H_2_O_2_), superoxide anion (O_2_•−), and singlet oxygen (^1^O_2_), is one of the earliest events upon pathogen recognition [21]. These molecules can directly damage nucleic acids, proteins, and lipids, but also function as secondary messengers in signaling cascades that activate defenses such as the hypersensitive response (HR), leading to localized cell death and restriction of pathogen spread [22]. Beyond their cytotoxicity, ROS, and particularly H_2_O_2_, mediate long-distance signaling and can activate plant systemic responses, including RNAi-based antiviral defense [23].

Plants have evolved a range of antioxidant systems to mitigate the harmful effects of excessive ROS levels. Catalase (CAT), one of the most effective enzymatic antioxidants, decomposes H_2_O_2_ into water and oxygen, thereby preventing oxidative damage [24]. Lipid peroxidation caused by ROS results in the formation of malondialdehyde (MDA), a well-established biomarker of oxidative membrane damage [25]. ROS can also oxidize DNA bases, generating mutagenic lesions such as 8-oxoguanine (8-oxoG), which require immediate repair via the base excision repair (BER) pathway. The enzyme 8-oxoguanine DNA glycosylase (OGG1) plays a central role in this process, safeguarding genome stability under both abiotic and biotic stress conditions [26].

The interplay between ROS production and detoxification, RNAi activation, and the regulation of both viral and host factors ultimately determines the trajectory of infection. Increasing attention is now being paid to how ROS signaling and antioxidant defenses integrate with molecular pathways controlled by both the virus and the host plant under combined stress conditions [27,28]. Conceptual models including multiple components and processes such as HSP70/HSP90, ROS-mediated RNAi, DNA repair through OGG1, and oxidative stress indicators such as MDA and 8-oxoG, may provide a bigger and more complex picture of plant–virus interactions. Yet, most of the available studies examined single-stress scenarios, whereas natural environments typically assume multiple overlapping stress factors.

The present study aimed at elucidation of the molecular and cellular responses of *Nicotiana benthamiana* to TBSV infection following preliminary heat treatment. The major focus was on the potential effect of the elevated temperatures on heat shock responses, disease phenotype, virus proteins’ accumulation, plant oxidative balance and antioxidant enzymes’ activity, expression of genes involved in antiviral defense, and DNA repair.

## 2. Results

### 2.1. Phenotypic and Physiological Effects of Combined Temperature and Viral Stress

**Figure 1:**
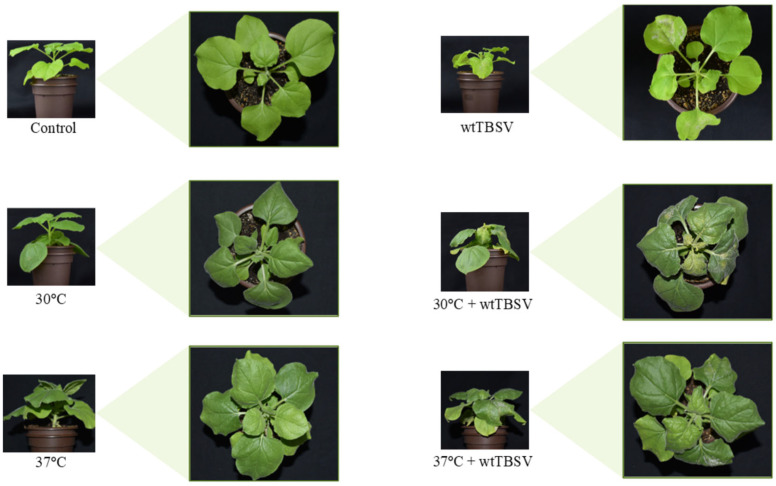
*N. benthamiana* plants at 7 dpi with wtTBSV and temperature stress. The illustration shows photographs from left to right: a control plant, a virus-inoculated plant grown at 25 °C (top row) similarly treated plants subjected to high-temperature stress of 30 °C (middle row) and 37 °C (bottom row). In plants subjected to combined stress, less pronounced symptoms were observed, such as wilting, leaf deformation, and reduced growth, except for local leaf necrosis. However, at 37 °C + wtTBSV, more intense chlorosis has been observed.

To assess virus concentration in plants and the impact of elevated temperature, N. benthamiana plants were initially grown under different temperature regimes, after which 30-day-old plants were inoculated with the wild-type tomato bushy stunt virus (wtTBSV). Symptoms of viral infection, such as leaf curling, stunted growth, and local chlorosis, were observed at 6–7 day post-inoculation (dpi) compared to control healthy mock-inoculated plants. In addition, under temperature stress, plants exhibited such phenotypic traits as leaf thickening and discoloration, shortened shoot length, and the development of additional lateral leaves. In case of combined stress, the symptoms of viral infection, such as the wilting of systemically infected leaves, were less pronounced compared to the action of single biotic stress (i.e., wtTBSV) (Figure 1).

**Figure 2:**
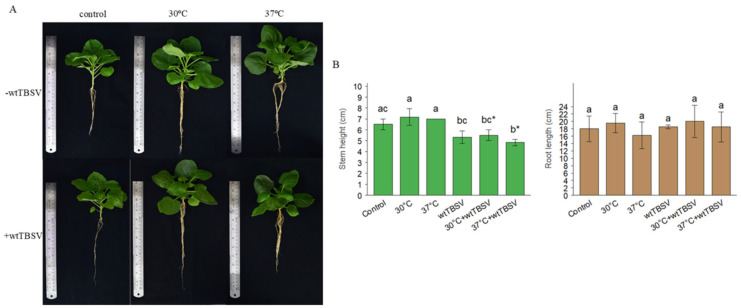
Combined effects of temperature and virus stresses on plant morphometric values. (**A**) Morphological appearance of Nicotiana benthamiana plants infected with wild-type TBSV (+wtTBSV) or mock-treated (−wtTBSV) under control conditions, 30 °C, and 37 °C. (**B**) Quantitative analysis of plant shoot height (left) and root length (right) under the same treatments. Statistically significant differences between different groups were supported by one-way analysis of variance (ANOVA) with Tukey’s HSD test, *p* < 0.001. Different letters (a, b, c) above the bars indicate statistically significant differences among treatments. Bars sharing the same letter are not significantly different. Asterisks (*) denote statistically significant differences at *p* < 0.05.

When conducting morphometric measurements of plant height and root length, a significant difference in plant height was observed as compared to the control plants. Morphometric analysis of N. benthamiana plants showed that the average shoot height was 6.5 ± 0.5 cm in the control group. Plants exposed to elevated temperature reached 7.17 ± 0.76 cm at 30 °C and 7.0 ± 0.0 cm at 37 °C, with no significant differences compared to the control (*p* > 0.05). Infection with wtTBSV reduced shoot height to 5.33 ± 0.58 cm, while combined stress conditions resulted in values of 5.5 ± 0.5 cm (30 °C + wtTBSV) and 4.83 ± 0.29 cm (37 °C + wtTBSV), with the latter significantly different from the control (*p* = 0.015). The average root length in the control group was 18.0 ± 3.46 cm. At 30 °C, root length was 19.5 ± 2.60 cm, and at 37 °C it decreased slightly to 16.17 ± 3.62 cm. wtTBSV infection alone resulted in a root length of 18.5 ± 0.5 cm. Under combined stress conditions, values of 20.0 ± 4.36 cm (30 °C + wtTBSV) and 18.5 ± 4.09 cm (37 °C + wtTBSV) were recorded. No significant differences were found among groups (*p* > 0.05) (Figure 2).

We have also measured the content of chlorophylls in the same groups of plants to show any possible physiological changes.

**Figure 3:**
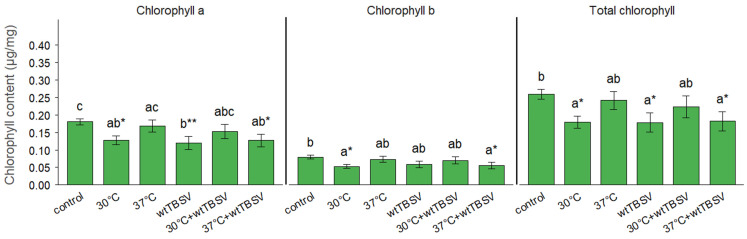
Combined effect of temperature and virus stresses on chlorophyll content in N. benthamiana leaves. Statistically significant differences between plant groups were supported using one-way analysis of variance (ANOVA) with Tukey’s HSD test, *p* < 0.001. Different letters (a, b, c) above the bars indicate statistically significant differences among treatments. Bars sharing the same letter are not significantly different. Asterisks (* and **) denote significant pairwise differences between specific treatments, with * *p* < 0.05, ** *p* < 0.01.

The content of chlorophyll A, B, and total chlorophyll varied depending on the conditions of combined stress at 30 °C and 37 °C and virus infection (Figure 3). As can be seen, virus infection generally led to the reduction of chlorophylls (less obvious for chlophyll B). Interestingly, the lowest values for chlorophylls A, B and total chlorophyll were demonstrated after the action of a single stressor, elevated temperature of 30 °C (but not 37 °C), the effect which was compensated by combined action of the virus infection.

### 2.2. Oxidative Stress and Antioxidant Responses Under Combined Stress Conditions

**Figure 4:**
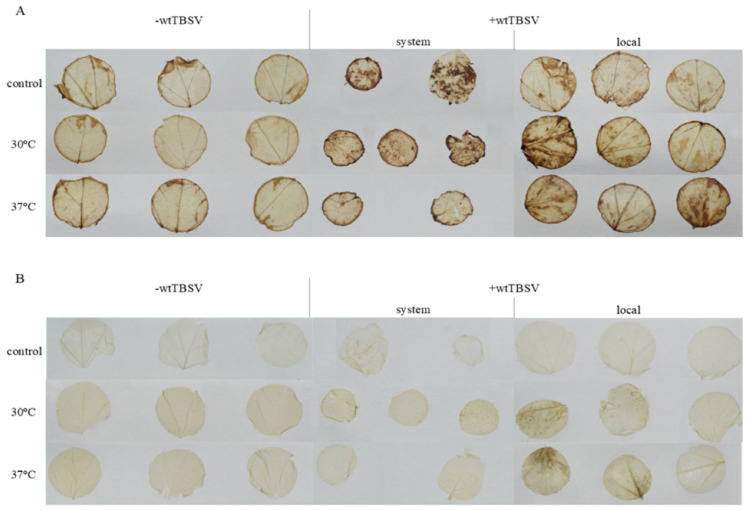
Combined effects of temperature and virus infection stresses on of hydrogen peroxide (H_2_O_2_) levels in N. benthamiana leaves. H_2_O_2_ accumulation was assessed by DAB staining, where brown color H_2_O_2_ presence. (**A**) Representative systemic and local leaves from plants either mock-inoculated (−wtTBSV) or inoculated with wtTBSV (+wtTBSV) and maintained under either control conditions, at 30 °C, or at 37 °C. (**B**) Corresponding unstained controls of systemic and local leaves under the same treatments, confirming that the observed coloration resulted from DAB staining and not from background pigmentation.

To assess oxidative stress in N. benthamiana leaves, 3,3′-diaminobenzidine (DAB) staining was used to visualize hydrogen peroxide (H_2_O_2_) accumulation. All control plants and plants which were not inoculated with the virus but subjected to heat stress, showed only weak DAB staining. Under combined stress, the staining was more intense as compared to the control leaves. These data indicate virus-induced oxidative stress. It was also noted that more intense oxidative stress occurred under combined stress at higher temperature (37 °C + wtTBSV) as compared to 30 °C + wtTBSV (Figure 4).

**Figure 5:**
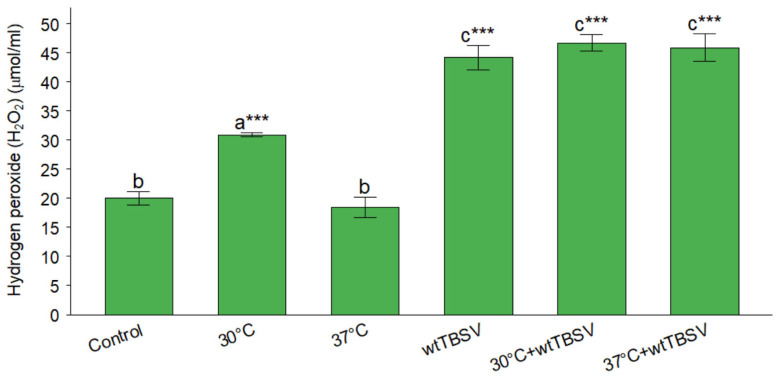
Combined effects of temperature and virus infection stresses on hydrogen peroxide levels in N. benthamiana leaves. Statistically significant differences between plant groups were supported by one-way analysis of variance (ANOVA) with Tukey’s HSD test, *p* < 0.001. Different letters (a, b, c) above the bars indicate statistically significant differences among treatments. Bars sharing the same letter are not significantly different, while bars with different letters are significantly different. Asterisks (***) denote highly significant differences relative to the control group at *p* < 0.001.

H_2_O_2_ quantification by spectrophotometry correlated with DAB staining data. At 30 °C, a moderate increase in H_2_O_2_ levels was observed as compared to control healthy plants, whereas at 37 °C no significant changes were observed (Figure 5). The most pronounced accumulation of H_2_O_2_ was observed in plants inoculated with the wtTBSV and under combined stress. The accumulation of H_2_O_2_ indicates a significant increase in oxidative stress, especially under combined stress.

**Figure 6:**
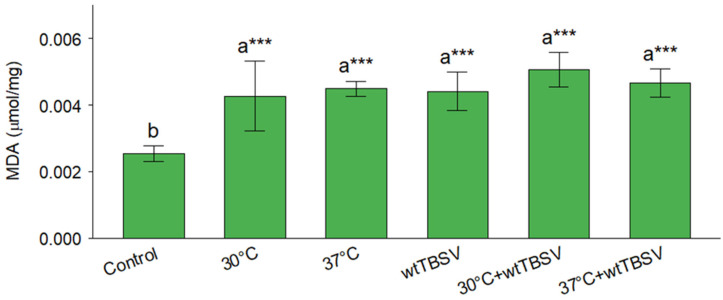
Combined effects of temperature and virus infection stresses on MDA content in N. benthamiana plants. Statistically significant differences between plant groups were supported by one-way analysis of variance (ANOVA) with Tukey’s HSD test, *p* < 0.001. Different letters (a, b) above the bars indicate statistically significant differences among treatments. Bars sharing the same letter are not significantly different, while bars with different letters are significantly different. Asterisks (***) denote highly significant differences relative to the control group at *p* < 0.001.

When assessing MDA content in plant leaves, the highest value was recorded for combined stress (30 °C + wtTBSV) conditions as compared to the lowest values in control healthy (mock-inoculated) plants. Compared to the control plants, the increase in MDA content in leaves of plants subjected to both stress factors was demonstrated regardless of the differences in stress (Figure 6).

**Figure 7:**
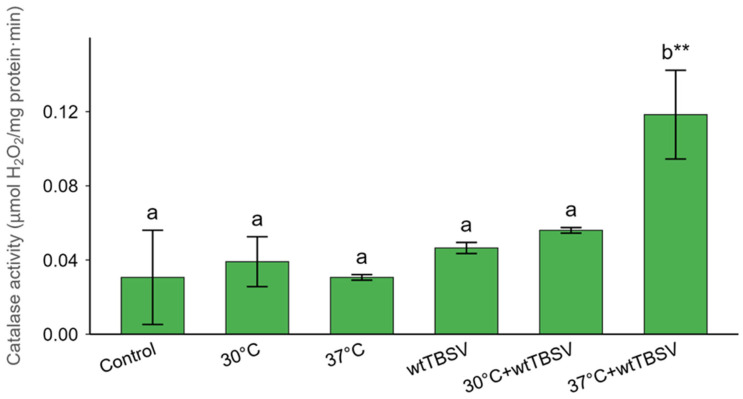
Combined effects of temperature and virus stresses on catalase activity (μM H_2_O_2_/mg protein·min) in N. benthamiana plants. Statistically significant differences between plant groups were supported by one-way analysis of variance (ANOVA) with Tukey’s HSD test, *p* < 0.001. Different letters (a, b) above the bars indicate statistically significant differences among treatments. Bars sharing the same letter are not significantly different, while bars with different letters are significantly different. Asterisks (**) denote highly significant differences relative to the control group at *p* < 0.01.

Spectrophotometric measurement of catalase activity revealed a slight increase in enzyme activity under thermal stress (30 °C, 37 °C) or viral infection when compared to the control. In contrast, a pronounced elevation of catalase activity was detected under the combined effect of elevated temperature (37 °C) and TBSV infection, indicating a strong activation of the antioxidant defense system in response to combined stress (Figure 7).

**Figure 8:**
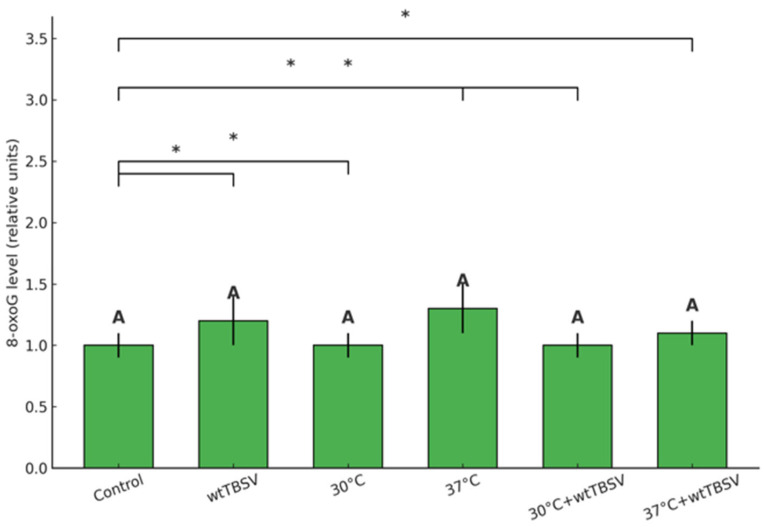
Combined effects of temperature and virus infection stresses on 8-oxoG level in N. benthamiana leaves. Statistically significant differences between plant groups were supported by one-way analysis of variance (ANOVA) with Tukey’s HSD test, *p* < 0.001. Letters A above the bars indicate statistically significant differences among treatments. Asterisks (*) denote significant pairwise differences between specific treatments, with * *p* < 0.05.

To determine the degree of oxidative DNA damage in N. benthamiana leaves, a quantitative ELISA assay of 8-oxoguanine (8-oxoG) content was performed. In the control group of plants, the 8-oxoG level averaged 1.00 ± 0.10 conventional units. In plants infected with the wild-type TBSV, a slight increase in the 8-oxoG level to 1.20 ± 0.20 was observed, similarly to the separate influence of temperature of 37 °C (1.30 ± 0.20). In groups with combined stress effect (30 °C + wtTBSV and 37 °C + wtTBSV), the 8-oxoG level did not statistically differ from the values of control plants, and was 1.00 ± 0.10 and 1.10 ± 0.10, respectively. A single exposure to 30 °C also did not influence the 8-oxoG level (1.00 ± 0.10). Overall, statistical analysis did not reveal significant differences between the groups (*p* > 0.05) suggesting that studied stress factors, either acting independently or in accord, did not induce noticeable oxidative DNA damage (Figure 8).

### 2.3. Effects of Combined Temperature and Viral Stress on the Accumulation of TBSV Virions and Host Stress-Responsive Markers

**Figure 9:**
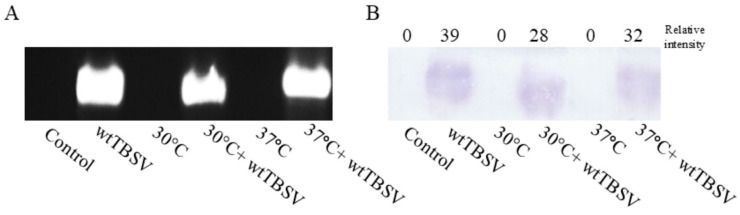
Combined effects of temperature and virus stresses on accumulation capsid protein CP41. (**A**) Visualization of TBSV genomic RNA on 1% agarose gel with ethidium bromide staining. (**B**) Western blot using primary antibodies to CP41.

To detect the accumulation of mature TBSV virions in infected Nicotiana benthamiana plants, we developed and applied an express analytical method integrating chromatographic, electrophoretic, and immunological detection techniques. The core of this method involves passing crude plant extracts through a hydroxyapatite-based column chromatography, which selectively retains viral particles based on their biochemical properties. The partially purified eluates were then subjected to agarose gel electrophoresis (1%), allowing for the separation and visualization of genomic viral RNA encapsidated in virions. Under UV illumination, distinct nucleic acid bands corresponding to virion-associated RNA were observed in samples from TBSV-infected plants, whereas no such bands were present in control samples. To confirm the viral origin of these nucleic acid bands, capillary transfer of the electrophoresed material onto a nitrocellulose membrane was performed, followed by immunoblotting using polyclonal mouse antibodies raised against the TBSV coat protein. Detection was achieved using secondary anti-mouse antibodies conjugated to alkaline phosphatase, and visualization was performed via NBT/BCIP substrate reaction. The results of horizontal gel electrophoresis showed the presence of viral particles (i.e., CP41) in all samples inoculated with the wtTBSV (Figure 9). However, under the combined stress (30 °C + wtTBSV) conditions, a reduction in virion-associated signal intensity was preliminarily observed under combined stress conditions, based on Western blot detection.

**Figure 10:**
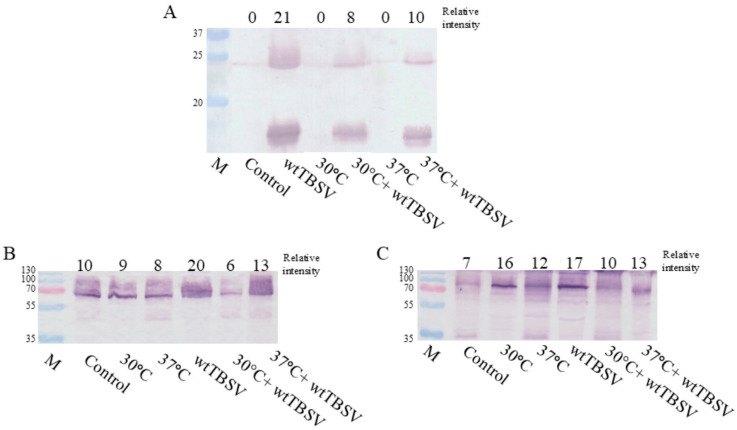
Combined effects of temperature and virus stresses on expression of P19, HSP70 and HSP90 in N. benthamiana leaves. (**A**) Western blot using primary antibodies to P19, (**B**) Western blot using primary antibodies to HSP70, (**C**) Western blot using primary antibodies to HSP90.

To determine the expression of host proteins (HSP70, HSP90), as well as the viral RNA interference suppressor protein (P19) in response to single and combined stresses, the Western blot was performed. To assess the accumulation of the viral factor P19, specific polyclonal rabbit P19-specific antibodies (Abcam, #ab106238, Cambridge, UK) were used. During the conducted experiments, an increase in virus content was observed under combined stress (37 °C + wtTBSV) conditions as compared to 30 °C + wtTBSV, where the expression of the P19 protein was even less pronounced as compared to single biotic stress. This is likely due to the high degree of damage caused by heat stress to plant cells, thereby reducing the effectiveness of the plant’s immune defense—RNA interference (Figure 10A). Additionally, the expression of heat shock proteins (HSP) (Agrisera, #AS08371 (HSP70), #AS08346 (HSP90), Vännäs, Sweden), which are proviral factors of the host cell and are directly involved in the replication of TBSV, was analyzed. Western blot for the HSP70 showed its increased level at elevated temperatures in both control and virus-inoculated plants (Figure 10B). HSP90 also showed increased expression, especially under combined stress (Figure 10C) conditions, which is consistent with studies on the involvement of these proteins in the spread of viral infection in plant cells.

**Figure 11:**
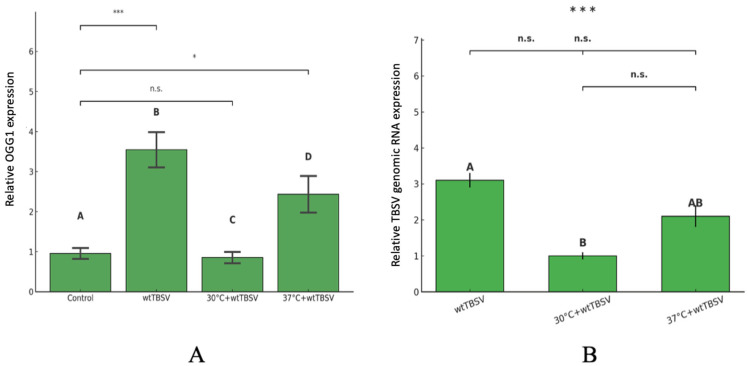
Combined effects of temperature and virus stresses on relative expression of OGG1 and accumulation of viral genomic RNA in N. benthamiana at 7 dpi. (**A**) Relative expression of OGG1 in control and TBSV-infected plants under control, 30 °C, and 37 °C conditions. (**B**) Relative levels of TBSV genomic RNA in infected plants under 30 °C and 37 °C conditions. Bars represent mean ± SD of three biological replicates. Different letters above bars indicate statistically significant differences between groups (ANOVA with Tukey’s HSD test, *p* < 0.05). Asterisks (* and ***) denote significant pairwise differences between specific treatments, with * *p* < 0.05, *** *p* < 0.001, n.s.: non-significant.

Also, we analyzed the expression level of host protein OGG1 and TBSV genomic RNA in N. benthamiana after either single treatment with wtTBSV or combined effect (temperature + virus) at different time intervals post infection (3, 5 and 7 dpi). Our results showed that, in seven days after inoculation with wtTBSV, the expression level of OGG1 was significantly higher as compared to control plants (3.2-fold, *p* = 0.0002), and also higher as compared to the combined stress group (37 °C + wtTBSV) (2.2-fold, *p* = 0.02). No significant difference was observed in 30 °C + wtTBSV group (*p* = 0.5) (Figure 11A). Similarly, in 7 days after inoculation with wtTBSV, the expression TBSV genomic RNA, based on qPCR amplification of the p33 coding region, was 3.1-fold higher as compared to healthy control plants (*p* = 0.0002). Combined stress treatment (37 °C + wtTBSV) resulted in a 2.1-fold increase in TBSV genomic RNA expression (*p* = 0.2), while TBSV genomic RNA expression in the combined stress group (30 °C + wtTBSV) was significantly lower than that in the single TBSV challenge group (*p* = 0.5) (Figure 11B).

## 3. Discussion

The impact of temperature stress on the development of viral infections in plants has been published. It is worth noting that some studies have reported plant tolerance to viral infections at elevated temperatures [29,30,31,32], while the others indicated that higher temperatures might lead to a weakened immune response in plants to the virus infection [33]. This underpins a varied nature of the optimal temperature depending on the plant species and virus origin. In this study, we have observed a decrease in the content of the wtTBSV in *N. benthamiana* plants at 30 °C.

Many plant species are highly sensitive to sudden temperature peaks, but gradual stepwise temperature elevation may facilitate plant adaptation to thermotherapy [34]. It has been shown that the use of day/night alternating temperatures during thermotherapy mitigates the negative impact of high constant temperatures on shoots in vitro, increases their survival and promotes better growth, which in turn increases the efficiency of virus elimination. In particular, Knapp et al., 2022 found that apple shoots subjected to heat treatment for 30 days at alternating temperatures of 38 °C/36 °C (day/night) demonstrated significantly higher survival rates compared to shoots treated at a constant temperature of 38 °C [35]. Similarly, strawberry shoots subjected to heat treatment at alternating temperatures of 42 °C/34 °C (day/night) for 50 days demonstrated higher survival rates, greater shoot length, and a higher proliferation index compared to shoots treated at a constant temperature of 37 °C [36]. The positive effect of alternating temperature regimes of thermotherapy on shoot survival has been noted for other woody crops. Thus, the use of alternating temperatures contributed to the effective elimination of viruses in peach [37], apple [38] and plums [39], where this approach ensured both higher rate of shoot survival and successful removal of virus infection.

Early studies have shown that elevated temperatures enhance RNA interference in plants, leading to the suppression of virus infection (and hence virus content) induced by tombusviruses, including cucumber necrosis virus (CNV) and TBSV [40,41]. In previous studies (Pantaleo & Burgyán, J., 2008) reduced accumulation of the cymbidium ringspot virus (CymRSV) was observed at elevated temperatures (28 °C), whereas at lower temperatures (20 °C), an increase in viral load was observed [42]. Additionally, temperature elevation up to 27 °C activated RNA interference leading to sharp decrease in the accumulation of turnip crinkle virus (TCV) and, consequently, milder symptoms of viral infection in *N. benthamiana* [32]. Despite the fact that only short-term exposure to elevated temperatures was used in a number of previous studies focused on combined stress and tombusviruses, in our work the symptoms of TBSV infection were alleviated after long-term temperature exposure. By 7 dpi, we observed less pronounced wtTBSV symptoms in *N. benthamiana* plants grown at 30 °C and 37 °C, such as mottling, leaf curling, necrosis, and chlorosis. Interestingly, under combined stress conditions (37 °C + wtTBSV), more pronounced wilting of systemic leaves and chlorosis of the lower tier leaves were observed as compared to other groups. This is consistent with the observations decribed in another study focused on tombusvirus infections in *N. benthamiana*, such as CymRSV and TCV [43].

Our results correlate with the findings of previous studies, where the height of plants significantly decreased in all three replicates under the influence of single abiotic and biotic stresses [44]. However, it should be noted that the degree of variation depended on the severity of stress factors. When studying the effect of high temperature on *Triticum aestivum* L., a decrease in the above-ground part of the plants was also observed, while the root length decreased only slightly [45]. In the studies by Obrępalska-Stęplowska et al., 2015, an increase in temperature when growing of *N. benthamiana* plants (27 °C/21 °C; day/night) was noted to enhance plant growth, whereas under combined stress conditions (temperature + virus), more efficient intracellular transport of peanut stunt virus was observed [46]. Relatively high temperatures accelerate ontogenetic development and significantly shorten the growth period in certain annual species, including major food crops such as *Solanum tuberosum* ssp. *tuberosum* L., *T. aestivum* L., *Oryza sativa* L., and *Solanum lycopersicum* L. [47,48,49,50].

The content of chlorophyll a, chlorophyll b, and total chlorophyll significantly differed in different plant groups under the influence of combined stress. The highest total chlorophyll content was observed in the control group at the treatment temperature of 37 °C. At the same time, the lowest total chlorophyll value was observed during a single effect of wtTBSV infection (Figure 3). Interestingly, earlier studies using *Arabidopsis thaliana* under high-temperature conditions (40/36 °C, day/night) showed a decrease in the content of chlorophyll a and b [51]. For *A. thaliana*, the optimal temperature is 20–25 °C, whereas for *N. benthamiana* it is 23–30 °C [52]. Assumingly, the plants adapt to higher temperatures by altering the levels of phytohormones and their signaling pathways. In particular, the adaptive response involves activation of abscisic acid (ABA)- and indole-3-acetic acid (IAA)-dependent signaling pathways involving SnRK2 kinases and auxin-dependent signaling via ARF factors [53,54,55,56]. As well as the pathways mediated by salicylic acid (SA) via the NPR1-dependent signaling pathway, jasmonic acid (JA) via the JA-JAZ-MYC signaling pathway and ethylene EIN-dependent signaling pathway provide stomatal regulation and osmotic adaptation [57,58,59]. In particular, the decrease in chlorophyll content in plant groups exposed to combined high temperature, water and salt stress may be related to physiological changes in leaves and stems, including inhibition of photosynthesis due to the combined stress [60]. In a study by Lei, R. et al., 2017, *Nicotiana tabacum* cv. *White Burley* plants inoculated with the M strain of cucumber mosaic virus had significantly lower chlorophyll a and b levels than healthy plants [61]. Chlorophyll a and b levels were also reduced in tomatoes (*S. lycopersicum*) infected with tobamoviruses tobacco mosaic virus (TMV), tomato mosaic virus (ToMV) and pepper mild mottle virus (PMMoV) [62]. The authors suggested possible activity of the enzyme chlorophyllase, which breaks down chlorophyll [63], and blocking of chlorophyll biosynthesis [64]. Rice stripe virus (RSV) can also suppress chlorophyll biosynthesis by inhibiting Mg-chelatase, which is responsible for the incorporation of magnesium ions into protoporphyrin IX in *O. sativa* var. *Japonica* [65]. It was also found that cucumber mosaic virus (CMV) Y satellite RNA (Y-sat) caused degradation of Mg-chelatase mRNA through RNA interference in *N. benthamiana* [66]. A decrease in chlorophyll content disrupts photosynthetic activity, weakens the formation of reactive oxygen species and promotes the successful spread of the virus.

Oxidative stress is a widely recognized response of plants to the influence of both abiotic and biotic factors. Increased accumulation of reactive oxygen species (ROS) was observed under the influence of various stressors, including high temperature, drought, and virus infections [67,68,69,70,71]. When staining the leaves with 3,3′-diaminobenzidine (DAB), the most intense accumulation of hydrogen peroxide (H_2_O_2_) was observed under the combined stress conditions (temperature + virus), especially at the temperature of 37 °C (Figure 4). In this case, wtTBSV-inoculated plants and plants subjected only to high temperature both showed moderate staining as compared to control healthy plants, indicating the absence of significant H_2_O_2_ accumulation when exposed to abiotic and biotic stress separately. Presumably, this is related to early activation of plant’s antioxidant system, including both enzymatic and non-enzymatic components. Interestingly, under combined stress, the systemic leaves of the plants did not stain as intensely as the leaves of wtTBSV-inoculated plants. Despite virus infection or thermal treatment, plants effectively prevented excessive accumulation of H_2_O_2_ and leaf necrosis, maintaining oxidative homeostasis.

Quantitative determination of hydrogen peroxide content by spectrophotometry confirmed the results of DAB staining. The highest accumulation of H_2_O_2_ was observed under the combined effect of wtTBSV and high temperature (Figure 5). Earlier studies using the DAB staining method to measure H_2_O_2_ revealed the accumulation of ROS under biotic and abiotic stresses [72,73,74], and also when measuring the level of H_2_O_2_ using spectrophotometry [75,76,77,78,79]. Interestingly, at high temperatures, there is a rapid accumulation of H_2_O_2_, however, after recovery, the same level of H_2_O_2_ was not observed in the plants [80]. These data confirm the involvement of H_2_O_2_ in the initiation of the cellular immune response and the suppression of viral infection in plants.

MDA formation is used as a common indicator of the degree of lipid peroxidation resulting from oxidative stress. In our work, we observed an increase in MDA levels in response to stress factors such as higher temperature and wtTBSV infection (Figure 6). Other researchers, when studying the effect of Cd, also observed an increase in MDA levels in roots and leaves of *Pistia stratiotes* plants, indicating peroxidative lipid membrane damage [81]. MDA levels also increased under combined stress caused by temperature and drought in *Hordeum vulgare* [82]. Virus infections induced by potato virus Y (PVY), potato leafroll virus (PLRV), and potato virus X (PVX) also led to an increase in MDA levels in tubers of *S. tuberosum* [83]. Similarly, Moreover, bean yellow mosaic virus in faba beans [84], maize dwarf mosaic virus (MDMV) infection in sweet corn *Zea mays* cv. *saccharata* var. Honey Koern [85], and pepper mild mottle virus (PMMoV-I) infection in *N. benthamiana* [77] all stimulated an increase in MDA levels. Lipid peroxidation causes disruption of the functional and structural integrity of biological membranes, increasing plasma membrane permeability, K^+^ ion leakage, amino acid oxidation, and ultimately leading to cell death [86]. An increase in MDA levels is also indicative of possible functional losses—disruption of cell membrane permeability and downregulation of photosynthetic activity leading to morphological damages to plant leaves [87]. This is consistent with the observed decrease in chlorophyll content and morphological signs in plants subjected to combined exposure of both stressors.

Under conditions of elevated reactive oxygen species (ROS) accumulation, plants initiate antioxidant defense mechanisms involving enzymes that specifically mitigate oxidative damage. Catalase (CAT) is a key enzyme responsible for the detoxification of hydrogen peroxide (H_2_O_2_), a prominent ROS formed under diverse abiotic and biotic stressors [88]. However, data concerning catalase activity under complex stress conditions remain fragmented and often species- or tissue-specific. Previous studies have demonstrated that pretreatment of virus-infected potato shoots with salicylic acid increased survival rates following thermotherapy (42 °C for 30 days) and enhanced the efficiency of Potato virus X (PVX) elimination from 75% to 98% across seven tested genotypes [89]. Interestingly, this treatment was associated with a decrease in catalase activity and a concomitant rise in H_2_O_2_ content, suggesting a signaling role of H_2_O_2_ in stress adaptation [90].

In our study, catalase activity was highest in TBSV-infected plants exposed to 37 °C. However, this did not correspond with a reduction in H_2_O_2_ levels; on the contrary, hydrogen peroxide remained elevated (Figure 7). Although catalase activity was significantly upregulated in TBSV-infected plants at 37 °C, this increase did not coincide with a reduction in hydrogen peroxide levels. This apparent contradiction likely reflects a compensatory antioxidant response to excessive ROS accumulation under combined viral and heat stress, rather than efficient detoxification. It is well established that under severe oxidative stress, plants activate antioxidant enzymes such as catalase to mitigate ROS damage. However, when ROS production exceeds the scavenging capacity of these enzymes, H_2_O_2_ levels can remain elevated despite increased catalase activity [91,92]. Similar phenomena have been observed under high temperature stress, where ROS generation outpaces detoxification mechanisms, even when catalase and other antioxidants are activated [93]. Moreover, viral infections themselves can dysregulate redox balance, contributing to persistent H_2_O_2_ accumulation despite elevated catalase activity [94].

Such discrepancies between catalase activation and ROS accumulation have been observed in multiple plant systems. In *Zostera marina*, H_2_O_2_ and malondialdehyde (MDA) levels increased under both cold and heat stress, while catalase expression was induced only under cold conditions and suppressed at high temperatures [88]. In *Hordeum vulgare*, heat-induced H_2_O_2_ accumulation was tissue-specific, with elevated levels in roots but not in leaves [95]. Likewise, long-term heat stress in *Arabidopsis thaliana* led to sustained H_2_O_2_ accumulation despite increased CAT enzymatic activity [96].

Unlike the findings of Yergaliyev et al. (2016), who reported a significant rise in CAT activity following wtTBSV infection, our data show that catalase activity remained unchanged compared to controls. This divergence may reflect the action of regulatory mechanisms that suppress antioxidant responses under combined stress conditions [97].

Additional factors may involve thermal destabilization of catalase itself. In *Vigna mungo*, the loss of CAT activity at elevated temperatures was attributed to conformational instability of its tetrameric structure [98]. Similarly, in potato, CAT activity was stable at temperatures below 20 °C but declined markedly at 50 °C and above [89]. In *Nicotiana tabacum*, seven catalase isoforms have been identified, with NtCAT5 and NtCAT6 predominantly expressed in roots and upregulated under cold stress conditions [90].

Taken together, the persistent accumulation of H_2_O_2_, despite catalase activation, suggests that ROS production exceeds detoxification capacity under the combined influence of heat and viral infection. Organ-specific regulation, differential expression of catalase isoforms, and the potential accumulation of additional ROS types may further contribute to redox imbalance. The concurrent elevation of MDA levels under multiple stress conditions supports the notion of a broader oxidative dysfunction in these plants.

Accumulation of ROS in cells causes subsequent base damage in DNA, forming 8-oxoguanine. According to recent studies, guanine is the most vulnerable nucleotide to ROS attack in DNA, and the formation of 8-oxoguanine is considered to be the most common type of oxidative damage to nitrogenous bases. In this regard, 8-oxoguanine is widely used as a reliable biomarker of oxidative stress and DNA damage [99,100,101]. In our study, 8-oxoG levels were not statistically different from control plants (Figure 8). This may indicate a change in the intensity of oxidative stress or activation of plant immune defense, including the repair and antioxidant systems of plants.

Early studies have shown that significant structural and functional changes occur in virus particles when temperatures rise above 37 °C. In particular, there is a breakdown of hydrogen and disulfide bonds in coat proteins, a rupture of covalent phosphodiester bonds in nucleic acids, and selective inhibition of viral replicase activity. These processes are accompanied by a change in pH and ionic strength of the cellular contents, an increase in the activity of lytic enzymes, and competition between viral RNA and plant mRNA for ribosomal binding sites [102]. When analyzing the content of coat protein TBSV-CP41 (as an indirect indicator of the potential number of progeny virions produced) at 7 dpi, a decrease in virion accumulation in plant cells was observed under high-temperature conditions of 30 °C and 37 °C (Figure 9). These results indicate that elevated temperature may negatively regulate the assembly of TBSV viral particles in the cell cytoplasm. The results are consistent with the study [103] where elevated temperatures may result in decreased efficiency of potato leafroll virus (PLRV) infection in *S. tuberosum*.

Several studies have shown that exposure to high temperatures enhances RNA silencing induced by cymbidium ringspot virus (CRSV) [42] and potato virus X (PVX) [104] in tobacco. Recently, Liu et al., 2016 found that heat treatment of pear shoots infected with apple stem grooving virus (ASGV) resulted in a significant reduction in viral RNA content accompanied by increased levels of virus-induced small interfering RNA (vsiRNA) [105]. Thermotherapy appears to stimulate vsiRNA biogenesis and reduce viral RNA accumulation by regulating the expression of key genes involved in RNA interference mechanisms [106]. In addition, it is known that microRNAs (miRNA) are also involved in the regulation of genes associated with plant resistance to viral infections [107]. The enhancement of RNA interference by high temperatures is currently considered one of the most compelling mechanisms for increasing the effectiveness of thermotherapy in targeting virus infections [108].

It is known that tombusviruses encode RNA interference suppressor proteins, thereby effectively neutralizing one of the main pathways of plant immune responses [109,110,111,112,113]. In the case of TBSV, P19 protein is one of the key factors in suppressing cellular immune defense. It has a high affinity for vsiRNAs and prevents their incorporation into the RNA-induced silencing complex (RISC), thereby effectively blocking the RNAi mechanism [114,115]. Under combined stress conditions (37 °C + wtTBSV), the accumulation of P19 protein was significantly reduced compared to plants inoculated with wtTBSV but not subjected to the heat stress (Figure 10A). This indicates the possibility of suppressing viral infection in plant cells through thermal treatment, which induces increased expression of RNAi genes. The results correlate with the studies by Nadhan et al., 2021 noting the early activation of RNAi in response to virus infection, which significantly suppressed virus load at the early stages of virus infection [116].

Moreover, TBSV, like many other (+)RNA plant viruses, actively utilizes host cellular factors. Heat shock proteins (HSP), particularly HSP70, play a key role in the formation of virus replication complex, where it interacts with virus protein P33 and activates viral RNA-dependent RNA polymerase (RdRp), ensuring efficient replication of the virus. Besides HSP70, plant viruses can also recruit other cellular chaperones, including HSP90. HSP90 is involved in the stabilization and activation of viral RdRp. Together with CDC37, it forms a complex with the viral RNA-dependent RNA polymerase P92, which is necessary for the initiation of viral genome replication [117,118,119]. The role of HSP proteins in host cells is very important, as they are involved in protein folding, prevent the aggregation of denatured proteins, and maintain cellular homeostasis during stress factors [120]. Analysis of heat shock protein expression showed that HSP70 and HSP90 are expressed both at high temperatures and during wtTBSV viral infection. However, under combined stress conditions, a decrease in HSP70 expression was observed, suggesting activation of plant antioxidant system, as previously described, and suppression of wtTBSV replication (Figure 10B, C). Earlier studies have identified an increase in HSP levels under the single effect of virus infection, and HSP expression under combined stress of high temperature and taxonomically diverse viruses, such as CNV, TYLCV, RSCNMV, TMV, PVY [121,122,123,124,125,126,127].

(+)RNA viruses depend on cellular membranes for their replication [128]. These cellular membranes concentrate membrane-associated virus replication proteins, forming a platform for the assembly of virus replicase complexes (VRCs) and providing protection from cellular nucleases and proteases [129]. TBSV encodes two major replication proteins, P33 and P92, which function together in VRC formation [130]. Our results demonstrated that relative viral RNA levels (based on qPCR amplification of the p33 coding region) was significantly higher in wtTBSV infection compared to combined stress (Figure 11B). As shown previously by Oster et al., mutations in the P33 ORF resulted in suppression of viral RNA replication, confirming its key role in VRC initiation and assembly [131]. Under combined stress conditions (temperature + virus), a significant decrease in P33 expression was observed, especially at 30 °C + wtTBSV. A similar decrease was demonstrated by Pogany et al., 2008 where the temperature-sensitive mutant (ts) of Ssa1p lost its ability to mediate TBSV VRC assembly in vitro at 30 °C, whereas only a partial decrease in activity was observed in wild-type Ssa1p [132]. This indicates that P33 is a temperature-sensitive virus protein, which is consistent with our results.

Elevated H_2_O_2_ and MDA levels observed during virus infection and high temperature indicated accumulation of ROS in plants. ROS cause various DNA damages including oxidized nucleobases, apurinic/apyrimidinic (AP) sites, and DNA strand breaks [133]. To remove oxidized bases and prevent mutations, as well as maintain genomic stability, cells employ efficient DNA repair systems, including base excision repair (BER) [134]. One of the key components of BER is the OGG1 gene encoding a DNA glycosylase/lyase involved in the removal of oxidized purine bases [135,136,137]. The data obtained showed that at 7 dpi, the expression level of OGG1 remained significantly higher in case of single wtTBSV infection (no heat treatment) as compared to control plants (Figure 11A). This may indicate the activation of the BER pathway and efficient repair of DNA damage, preventing the accumulation of 8-oxoG. Under 37 °C + wtTBSV combined stress conditions, OGG1 expression was reduced as compared to wtTBSV infection only, while under 30 °C+ wtTBSV the expression level did not differ from that in control plants (Figure 11A). Despite high H_2_O_2_ and MDA levels, it is likely that the low OGG1 expression under these conditions was due to either a reduced need for repair system’ activation because of the suppressed virus replication or the prevalence of alternative antioxidant and defense mechanisms such as RNAi. Chen et al., 2012 showed that overexpression of AtOGG1 in *A. thaliana* resulted in decreased 8-oxoG levels in seeds, improved germination and increased tolerance to various types of abiotic stress such as high temperature, salt, osmotic and oxidative stress [138]. Elevated expression of MtOGG1 was also observed in *Medicago truncatula* under Cu^2+^-induced oxidative and osmotic stress [139]. Thus, OGG1 expression reflects repair mechanisms under conditions of wtTBSV viral infection and combined stress (temperature + virus), and can be considered as one of the molecular markers of the involvement of the BER pathway in the cellular response of plants to oxidative DNA damage.

In summary, this study provides new evidence that the interaction between moderate heat stress and TBSV infection in *Nicotiana benthamiana* induces a distinct, non-additive physiological response. The observed divergence between catalase activation and sustained H_2_O_2_ accumulation under combined stress suggests a functional exhaustion of the antioxidant system and impaired redox buffering capacity. Our analysis further demonstrates that the expression of key viral (P19, P33) and host (HSP70, HSP90, OGG1) components is tightly modulated by the temperature context, emphasizing the importance of environmental conditions in shaping virus–host dynamics. These findings collectively underscore that plant responses to overlapping stress factors are governed by integrated and often antagonistic signaling networks. By dissecting these responses at both the molecular and biochemical levels, our work advances the mechanistic understanding of stress interplay in plant-virus interactions and offers a conceptual framework for future studies aimed at improving crop resilience under multifactorial environmental challenges.

## 4. Materials and Methods

### 4.1. Plant Material, Growth Conditions, and Virus Inoculation

*Nicotiana benthamiana* plants were grown in soil with vermiculite (3:1) with a 16-h light period (7500 lux) at 25 °C for 15 days at 65% humidity. Beginning on the 20th day of growth, plants were subjected to a 13-day heat stress regime. The control group was maintained at 25 °C, while experimental groups were exposed to alternating cycles of high temperature (30 °C or 37 °C) for 30 h, followed by recovery at 25 °C for 42 h, with a final recovery phase of 24 h (Table 1). This design enabled simulation of recurrent heat stress to assess plant physiological responses.

Inoculation of 30-day old plants with wtTBSV was carried out by rubbing leaves of the third tier with 0.001% carborundum (Sigma-Aldrich, #409212, St. Louis, MO, USA). wtTBSV was mixed with a phosphate buffer (3,3 mM Na_2_HPO_4_, (Sigma-Aldrich, #7558794, St. Louis, MO, USA), 6.7 mM NaH_2_PO_4_, (Sigma-Aldrich, #7558807, St. Louis, MO, USA), pH 6.9–7.0) in a 1:3 ratio, respectively, and on the 7th day post-inoculation (dpi), morphometric measurements of shoots and roots were taken. To maintain the wild-type genome of tomato bushy stunt virus, plasmid DNA constructs pTBSV100 were employed. These plasmids contained a T7 promoter, LacZ-α selection marker, an ampicillin resistance gene (AmpR), and the full-length cDNA of wild-type TBSV. The plasmids were propagated in *Escherichia coli* strain DH5α for stable maintenance and amplification. The viral constructs were kindly provided by Professor Herman Scholthof (Texas A&M University, College Station, TX, USA), ensuring access to validated and previously described [130] molecular clones for experimental use.

### 4.2. Determination of Chlorophyll Content

The chlorophyll content was determined by the Arnon method [140] with modifications. In brief, 100 mg of plant leaves were homogenized in 2 mL of pre-cooled 80% ethanol, followed by centrifugation at 10,000 rpm for 10 min. Next, 8 mL of 80% ethanol was added to the supernatant in the dark, and chlorophyll content was measured on a Multiskan SkyHigh (Thermo Fisher Scientific, Waltham, MA, USA) spectrophotometer at wavelengths of 664 nm and 647 nm. To calculate the levels of chlorophyll a, chlorophyll b, and total chlorophyll in µg/mL, the following formulas were used [141]:

$$\begin{array}{ll} Chlorophyll\;a= & 13.36\times Abs_{\left\{664\right\}}-5.19\times Abs_{\left\{647\right\}}Chlorophyll\;b\\ & =27.43\times Abs_{\left\{647\right\}}-8.12\times Abs_{\left\{664\right\}}Total\;chorophyll\\ & =\left(13.36\times Abs_{\left\{664\right\}}-5.19\times Abs_{\left\{647\right\}}\right)\\ & +\left(27.43\times Abs_{\left\{647\right\}}-8.12\times Abs_{\left\{664\right\}}\right) \end{array}$$

### 4.3. ROS Determination

To determine the concentration of H_2_O_2_ in vivo, control and inoculated plant leaves were subjected to vacuum infiltration in 5 mM DAB (3,3′-diaminobenzidine tetrahydrochloride, Sigma-Aldrich, #2310189, St. Louis, MO, USA) and 10 mM Na_2_HPO_4_ (Sigma-Aldrich, #7558794, St. Louis, MO, USA) (pH 3.8) for 15 min. As a control, the plant leaves were incubated in 10 mM Na_2_HPO_4_ without the addition of DAB. After incubation, the leaves were transferred to a solution of ethanol/acetic acid/glycerin in a 3:1:1 ratio and boiled for 15 min in a water bath.

Determination of H_2_O_2_ concentration in vitro was performed using the method [142]. 100 mg of leaves were homogenized in 1 mL of 0.1% TCA on ice. Then the samples were centrifuged at 12,000 rpm for 10 min at 4 °C. Next, a 1:1 volume of 10 mM phosphate buffer (pH 7.5) and 1:2 volume of 1 M KI (Sigma-Aldrich, #7681110, St. Louis, MO, USA) were added in the dark. The absorption of the samples was measured at 390 nm on the Multiskan SkyHigh spectrophotometer (Thermo Fisher Scientific, Waltham, MA, USA).

y=a∗x+b

### 4.4. Determination of Malondialdehyde (MDA) Content

The MDA levels in the leaves were determined as described by Srivastava et al., 2017, with minor modifications [143]. 100 mg of fresh leaves were homogenized with a pestle and mortar in a chilled phosphate-buffered saline containing 10% trichloroacetic acid (TCA, Sigma-Aldrich, #76039, St. Louis, MO, USA). After centrifugation at 3000 rpm for 10 min, collected supernatant was incubated at 95 °C for 45 min in a water bath with an equal amount of 0.25% thiobarbituric acid (TBA, Sigma-Aldrich, #504176, St. Louis, MO, USA) solution. The samples were then cooled on ice and subsequently centrifuged at 10,000 rpm for 15 min. The supernatant was transferred for further measurement on a Multiskan SkyHigh spectrophotometer (Thermo Fisher Scientific, Waltham, MA, USA) at wavelengths of 532 and 600 nm. To calculate MDA in μmol/mL, the following formula was used [144]:

$$MDA(uM/mL)=6.45\times ({\mathrm{Abs}}_{532}-{\mathrm{Abs}}_{600})-0.56\times {\mathrm{Abs}}_{450}$$

### 4.5. Determination of Catalase (CAT) Activity

The measurement of catalase enzymatic activity was carried out as follows [145]: 100 mg of *N. benthamiana* leaves were homogenized in 0.1 M phosphate buffer (pH 6.8–7.0) at a 1:3 ratio and centrifuged at 15,000× *g* at 4 °C for 20 min. To prevent the loss of enzymatic activity, the reaction was carried out no later than two h after sample preparation. The enzymatic activity of catalase was determined in cuvettes using the Multiskan SkyHigh spectrophotometer (Thermo Fisher Scientific, Waltham, MA, USA). Before measuring catalase activity, all solutions were in a water bath warmed to 30 °C. In a 3.5 mL quartz cuvette, 3.27 mL of reaction buffer (50 mL of 0.1 M phosphate buffer (pH 6.8–7.0), 30 µL of 33% H_2_O_2_, #131077, PanReac AppliChem, Darmstadt, Germany) was added, and the reaction was initiated by adding 230 µL of the sample. The mixture was shaken quickly and measured at a wavelength of 240 nm every 10 s for 2 min. In the control sample, an equal volume of buffer without the sample was added. In both the experimental and control groups, the determination was carried out in three biological replicates. The obtained data are used to calculate the rate of decrease of the maximum absorption peak over the time interval corresponding to the linear section of the curve at a wavelength of 240 nm (tg = ΔD/t). To transition from the trigonometric function (tangent) to absolute units of enzyme activity, the formula was used:

$$CA=\left(tg_{0}-tg_{k}\right)\times V_{n}/(0.036\times Cᵦ)$$

where CA—catalase activity, μm H_2_O_2_/mg protein × min; tg_o_—ΔD/t for the experimental sample; tg_k_—ΔD/t for the control sample; V_n_—Total volume of the sample (3 mL); 0.036 mM^−1^·cm^−1^—Coefficient of extinction of H_2_O_2_; Cᵦ—Protein content in the sample, mg. The amount of soluble protein in the samples was determined by the Bradford method [146].

### 4.6. Determination of 8-Oxoguanine

To extract total DNA, 50 mg of *N. benthamiana* leaves were ground in 800 µL of preheated CTAB buffer (3% CTAB, (Sigma-Aldrich, #57090, St. Louis, MO, USA), 1.4 M NaCl, (Sigma-Aldrich, #7647145, St. Louis, MO, USA), 0.8 M Tris-HCl (Sigma-Aldrich, #1185531, St. Louis, MO, USA) (pH 8.0), 50 mM EDTA (Sigma-Aldrich, #6381926, St. Louis, MO, USA) (pH 8.0), 0.3% β-mercaptoethanol) (Sigma-Aldrich, #M6250, St. Louis, MO, USA)) at 65 °C. The samples were incubated at 65 °C for 1 h. Next, an equal volume of chloroform:isoamyl alcohol (24:1) (Sigma-Aldrich, #P3803, St. Louis, MO, USA) was added. The samples were centrifuged at 26,500× *g* for 15 min, and afterwards the supernatant was transferred into a new tube. Then, half the volume of 6 M NaCl, 1/10 of the initial volume of 3 M NaAc (Sigma-Aldrich, #127093, St. Louis, MO, USA) (pH 4.0), chilled isopropanol, (Amresco, #67630, Solon, OH, USA), and 5 µL of linear polyacrylamide (LPA, Thermo Fisher Scientific Baltics UAB, #AM9520, Waltham, MA, USA) were added, followed by incubation at −20 °C for 20 min, centrifugation at 26,500× *g* for 5 min, and removal of the supernatant. To remove impurities, 1000 µL of 70% ethanol was added. Next, the tubes with the centrifugate were dried at room temperature and resuspended in 50 µL of 1xTE buffer (10 mM Tris-HCl (pH 8.0), 1 mM EDTA). To completely dissolve the pellet, the samples were incubated at 50 °C for 1 h. The concentration of the isolated DNA was determined using a Qubit fluorometer (Thermo Fisher Scientific, Waltham, MA, USA) with #Q32850, Qubit™ dsDNA Broad Range. To determine the content of 8-oxoguanine in plant tissues, an ELISA was conducted according to the manufacturer’s protocol (QIAGEN, #4380-096-K69181, California, CA, USA), followed by the measurement of optical density at the wavelength of 450 nm using the Multiskan SkyHigh spectrophotometer (Thermo Fisher Scientific, Waltham, MA, USA).

### 4.7. Determination of Viral Particles in Leaves

To isolate viral particles, systemically infected leaves were ground with 10 mM phosphate buffer (3.3 mM Na_2_HPO_4_, 6.7 mM NaH_2_PO_4_, pH 6.8) on ice. Next, affinity chromatography on a hydroxyapatite matrix was performed according to Omarov et al., 2017 [147]. An express analysis of capillary transfer from agarose gel to nitrocellulose membrane was used to identify the virus coat protein P41. The procedure was carried out according to Omarov et al., 2019 [148], followed by the incubation of primary polyclonal mouse antibodies against the P41 coat protein and secondary anti-mouse antibodies conjugated with alkaline phosphatase. Next, the membrane was incubated with the NBT/BCIP (Rockland, #48239, Pennsylvania, PA, USA) substrate.

### 4.8. Westernblot

For protein extraction from N. benthamiana plants, a 1 × TE buffer (10 mM Tris-HCl, 1 mM EDTA) was used. The separation of proteins was carried out using a 15% polyacrylamide gel under denaturing conditions. Electrophoresis was conducted at 110 V, 50 mA, 5 W for 3 h in 1 × TGS buffer. To determine the expression of proteins P19, HSP70, HSP90 in the samples, a Western blot was used. The transfer was performed on the Turbo blot Semi Dry system (Bio-Rad Laboratories, Inc., Berkely, CA, USA). The transfer assessment was conducted using Ponceau S staining of the membrane. Blocking of the membrane was performed using 3% non-fat dry milk (Rockland, #B501-0500, Pennsylvania, PA, USA, 1 × TBS-Tween 20, Thermo Fisher Scientific, #003005, Waltham, MA, USA) at 25 °C, followed by washing with 1 × TBS-Tween 20 three times for 10–15 min. Incubation with primary antibodies was carried out for two h at 40 °C, followed by incubation with secondary antibodies conjugated with alkaline phosphatase. Protein detection was performed by incubating the membrane with the alkaline phosphatase substrate—NBT/BCIP for 3–5 min in the dark.

### 4.9. Extraction of Total RNA

The extraction of total RNA was carried out according to the manufacturer’s protocol using a commercial kit (#(NEB, #T2010S, Ipswich, UK). The concentration of total RNA was determined using a Qubit fluorometer with #Q10210, Qubit™ RNA BR (Thermo Fisher Scientific, Waltham, MA, USA).

### 4.10. RT-qPCR

cDNA synthesis was carried out according to the manufacturer’s protocol UltraqScript cDNA Supermix (#95217-9100, Quantabio, Beverly, MA, USA), followed by RT-qPCR (iTaq Universal SYBR Green Supermix, #1725120, BioRad, Hercules, CA, USA) according to the manufacturer BioRad CFX Opus 96. The RT-qPCR cycle consisted of 95 °C for 10 min, followed by 40 cycles (95 °C for 15 s and 56 °C for 1 min). As an endogenous housekeeping gene, GAPDH was used. The relative RNA expression level was calculated using 2 × (−ΔΔCt). Primers are listed in Table 2.

### 4.11. Statistical Analyses

One-way analysis of variance (ANOVA) was used to compare different plant groups. Data are presented as mean ± SEM. The following probability levels were adopted to assess statistical significance: *p* < 0.05; ** *p* < 0.01; *** *p* < 0.001; ns—*p* > 0.05. All data were analyzed using GraphPad Prism 9 software (GraphPad Software, Inc., La Jolla, CA, USA) and Image J software for membrane analysis. All experiments were performed in at least three biological and three technical replicates.

## 5. Conclusions

This research demonstrated that elevated temperature significantly affected the susceptibility of *N. benthamiana* to tomato bushy stunt virus infection. Thermal pre-treatment at 30 °C and 37 °C before inoculation attenuated the symptoms of viral infection and resulted in decreased virus content, as evidenced by decreased expression of coat protein CP41 and RNA interference suppressor P19. These changes indicate that temperature stress can activate plant defense mechanisms, including the antioxidant system and RNA interference, which together limit the development of viral infection. At the same time, the combined effect of TBSV and elevated temperature induced increased oxidative stress, which was manifested by the accumulation of H_2_O_2_ and MDA, as well as an increase in catalase activity. However, despite the pronounced accumulation of reactive oxygen species, the level of oxidative DNA damage, determined by the content of 8-oxoguanine, remained at the level of the healthy control plants. This may indicate either an efficient activation of the DNA damage repair system or secondary mechanisms preventing the critical consequences of stress. Indeed, the expression of the OGG1 gene, a key participant in the base excision repair (BER) pathway, increased during viral infection, but was reduced under the combined effect of virus and temperature, indicating variable activity of DNA repair mechanisms depending on the nature of stress. Interestingly, under the background of combined stress, a decrease in the expression of both viral factors (P33) and plant host factors, including HSP70 and HSP90, was observed, which probably additionally contributes to the inhibition of TBSV replication and virion assembly. Taken together, obtained data confirm that temperature stress is able to modify virus–host interactions such that the plant acquires temporary resistance to infection. This opens up new possibilities for the practical use of thermotherapy as a method of controlling phytopathogens, especially in closed soil conditions, where microclimate regulation is possible. The practical significance of this work is that temperature effects can be used not only to reduce the virus load, but also to stimulate endogenous plant defense mechanisms. Such data are important for the development of new approaches in the agro-industrial complex in the context of global climate change, where extreme temperatures are becoming an increasingly frequent stress factor.

## Figures and Tables

**Table 1:** Plant temperature stress simulation graph.

Phase	Time (h)	Control	1st group	2nd group
Hight temperature	30	25 °C	30 °C	37 °C
Recovery	42	25 °C	25 °C	25 °C
Hight temperature	30	25 °C	30 °C	37 °C
Recovery	42	25 °C	25 °C	25 °C
Hight temperature	30	25 °C	30 °C	37 °C
Recovery	42	25 °C	25 °C	25 °C
Hight temperature	30	25 °C	30 °C	37 °C
Recovery	42	25 °C	25 °C	25 °C
Hight temperature	30	25 °C	30 °C	37 °C
Recovery	24	25 °C	25 °C	25 °C

**Table 2:** Sequence of Primers Used for RT-qPCR.

Target Name	Sequence	Tm
GAPDH(At1g12900)	5′-3′F: agctcaagggaattctcgatg/5′-3′R: aaccttaaccatgtcatctccc	60
P33	5′-3′F: tgatttcgcaaccggagtga/5′-3′R: acccttaagttcccttgccg	59.7
OGG1	5′-3′F: gacctacatctcagccgtcg/5′-3′R: tgctaccttcggaccaacac	59.9

## Data Availability

The raw data from this study will be made available upon request to the corresponding author.

## Abbreviations

The following abbreviations are used in this manuscript:

TBSV	Tomato bushy stunt virus
CAT	Catalase
ROS	Reactive oxygen species
MDA	Malondialdehyde
H_2_H_2_	Hydrogen peroxide
HSP	Heat shock proteins
OGG1	8-oxoguanine DNA glycosylase
8-OxoG	8-oxoguanine
